# P-2055. County-Level Social Vulnerability Index and Infectious Disease Mortality Among Adults in the United States

**DOI:** 10.1093/ofid/ofaf695.2219

**Published:** 2026-01-11

**Authors:** Birgit Agyeiwaah Baah, Kwabena Asante Asabere, Aimee Eyram Eklu, Una Kanor, George Akwetey Junior, Wendy Priya Miranda, Frederick Dapaah-Siakwan

**Affiliations:** Altru Health System, Grand Forks, ND; Johns Hopkins Bloomberg School of Public Health, MPH/MBA Program, Baltimore, Maryland; Alliance Primary Care, Hoover, Alabama; Mount Sinai Hospital, Chicago, Chicago, Illinois; Albany Medical Center, Albany, New York; University of Ghana Medical Centre, Accra, Greater Accra, Ghana; Valley Children's Healthcare, Madera, California

## Abstract

**Background:**

Social vulnerability index (SVI) measures social determinants of health in United States (US) counties and has been used to assess the impact of social vulnerability on health outcomes including COVID-19 mortality. It is unknown if county-level SVI negatively impacts mortality from all infectious diseases (ID). We assessed the relationship between the SVI and ID-related age adjusted mortality rates (ID-AAMR) across US counties from 2018 to 2023.

**Figure:**
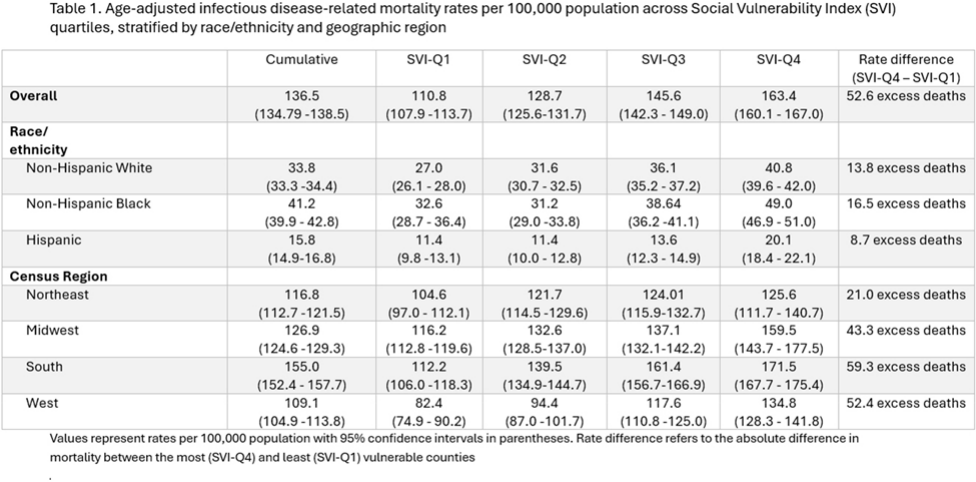

**Figure:**
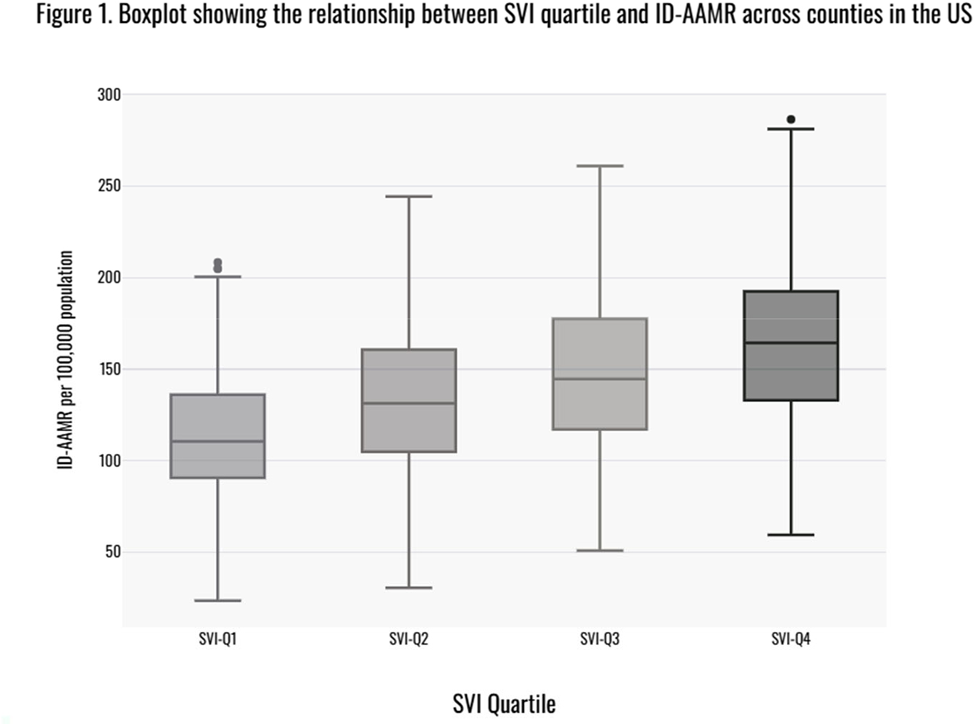

**Methods:**

We conducted a retrospective cross-sectional analysis of the multiple cause of death dataset from the CDC WONDER database. The ID-AAMR for all US residents aged ≥20 years who had any ID (ICD-10 codes A00-B99) listed on the death certificate were included. This was linked to the corresponding 2022 SVI score (range: 0 to 1; higher scores indicate greater vulnerability). The dataset was divided into quartiles based on SVI scores (0-0.25: 1^st^ quartile (SVI-Q1), least vulnerable, and 0.75-1 as SVI-Q4, most vulnerable). Counties with death counts < 20 were excluded. The ID-AAMR per 100,000 population and 95% confidence intervals (CI) were estimated for the overall population and stratified by race and census region. The exposure was SVI quartile. The outcome was the rate difference in ID-AAMR between SVI-Q4 and SVI-Q1 and was significant if the CIs did not overlap. We used negative binomial regression to estimate rate ratios (RRs) with SVI-Q1 as the reference. The RR ratios were significant if the CI excluded 1. The analysis was repeated with ID as the underlying cause of death.

**Results:**

Among 2,951 counties, the overall ID-AAMR per 100,000 was 136.5 (CI: 134.8–138.5). There was a stepwise increase in the overall ID-AAMR from 110.8 in SVI-Q1 to 163.4 in Q4 (RR 2.8, CI: 2.6 – 3.2), yielding an excess of 52.6 in SVI-Q4 (Figure 1). Similarly, SVI-Q4 was associated with excess ID-AAMR across all racial/ethnic groups and all geographic regions studied (Table 1) compared to SVI-Q1. When limited to ID as the underlying cause of death, the ID-AAMR increased significantly from 25.9 (CI: 25.1 – 26.8) in SVI-Q1 to 38.5 (CI: 37.5 – 39.5) in SVI-Q4 (RR 2.1; CI: 1.8 – 2.3).

**Conclusion:**

Counties with higher SVI were associated with increased ID-related mortality. Targeting public health efforts towards the most vulnerable could mitigate poor outcomes.

**Disclosures:**

All Authors: No reported disclosures

